# Supplementary material for: Sportspersonship orientation in the training process of young football players in different training contexts
Source: PLoS One. 2024 Oct 18;19(10):e0312167. doi: 10.1371/journal.pone.0312167 (PMC11488706; doi:10.1371/journal.pone.0312167)
Supplement: Attachment — Submitted filename: Response to Reviewer 1.docx [file pone.0312167.s001.docx]

**REBUTTAL LETTER**

Manuscript PONE-D-21-05952 [EMID:0e2927bc8a1101f0]

The development of respect in young athletes: A systematic review and meta-analysis

PLOS ONE

**Reviewer 1’s comments and suggestions for authors and details of the revisions and responses**

1.- The introduction of the article is too extensive. You need to shorten it.

We have made the change indicated.

2.- The goal of the article should be written in the past tense.

We have made the change indicated:

That is why the aim of this study was to analyse the evolution of sportspersonship orientation in the social sports schools of Fundación Real Madrid (FRM) and in association football clubs.

3.- The statistical analysis was done incorrectly, therefore the results presented in this study are not correct.

Taking into account the considerations of the reviewer, we consider that the statistical analysis carried out is correct, as this analysis responds to the objectives of the research. However, we have considered the reviewer's comment and made some changes in order to carry out a more complete statistical mathematical approach since we have carried out a multifactorial analysis.

Specifically, a basic descriptive analysis of central trend and dispersion of each dimension was performed. To determine whether there were differences between the two contexts (FRM vs. federated Clubs), the T-Student contrast test was used for independent samples. In cases where the data showed significant differences (p .05), effect size was calculated using standardized mean differences (size of Cohen effect). To examine whether there was an interaction effect between Context (FRM vs. Clubs) and Age Categories (U10, U12, U14, U16), a two-factor ANOVA (2x4) was performed. An analysis of estimated marginal means was performed; simple main effects were compared by post hoc testing with Bonferroni's confidence interval adjustment.

**THANK YOU** for your comments and suggestions.
